# Urinary 6-sulfatoxymelatonin level and breast cancer risk: systematic review and meta-analysis

**DOI:** 10.1038/s41598-017-05752-9

**Published:** 2017-07-13

**Authors:** Jing Xu, Lei Huang, Guo-Ping Sun

**Affiliations:** 1 Department of Medical Oncology, the First Affiliated Hospital of Anhui Medical University, Hefei, China; 2 Department of Gastrointestinal Surgery, the First Affiliated Hospital of Anhui Medical University, Hefei, China

## Abstract

6-Sulfatoxymelatonin (aMT6s) is the main metabolite of melatonin in urine, and is a reliable surrogate biomarker reflecting the blood melatonin concentration. This meta-analysis assessed the association between urinary aMT6s level and BC incidence. The electronic databases PubMed, EMBASE, Cochrane Library, and Web of Science were searched. Risk ratios (RRs) were adopted to estimate the relative BC incidence. A total of 7 prospective case-control publications were included, and 6 of them were distinct studies. Pooled analysis of data from the 6 studies involving 1824 women with incident BC and 3954 matched control participants with no overlapping of subjects among studies indicated no significant association between the highest levels of urinary aMT6s and the incidence of BC (RR = 0.97, 95% CI, 0.88–1.08, *P* = 0.56). Negative associations were observed in postmenopausal women (RR = 0.88, 95% CI, 0.75–1.02, *P* = 0.10), estrogen receptor positive BC (RR = 0.83, 95% CI, 0.64–1.07, *P* = 0.15), and studies using 12-hour overnight urine (RR = 0.81, 95% CI, 0.61–1.07, *P* = 0.13), all with borderline significances. Lag time or invasive degree did not interfere with the results. There was no evident publication bias detected by the Egger’s test and the funnel plot. Conclusively, the current evidence did not support a significant association between urinary aMT6s level and BC risk.

## Introduction

Breast cancer (BC) is one of the most common malignancies and a leading cause of cancer-related mortality among women worldwide^1, 2^. Night-shift work has been suggested to be a risk factor for BC and was classified as a group 2 A carcinogen by the International Agency for Research on Cancer (IARC)^3^. Women who ever had night-shift work had a significantly increased risk of BC, compared to those who had normal sleep duration. Melatonin (N-acetyl-5-methoxytrptamine) is secreted primarily by the pineal gland in humans. It has an intricate role in chronobiology, regulating the circadian rhythm^4^. The long-term disruption of decreased nocturnal melatonin production in night-shift workers has been associated with modestly increased risk of BC and other cancer types^5^.

There has been no definite explanation of the mechanism by which melatonin affects the development of BC. One hypothesis suggests that a lower level of melatonin secretion at night may lead to increased estrogen levels^6^. It is likely to impact estrogen metabolism through the selective estrogen receptor modulator (SERM) and the selective estrogen enzyme modulator (SEEM) activities, resulting in increased turnover of breast epithelial stem cells, and in thus subsequently raised risk of malignant transformation^7^. Physiological concentrations of melatonin have been demonstrated to down-regulate the aromatase expression in the MCF-7 human BC cell lines, showing a synergistic anti-proliferative effect with tamoxifen^8^. Melatonin also appears to directly promote apoptosis^9^ and inhibit angiogenesis^10^. In addition, it seems to have immunopotentiating and oncostatic effects by increasing the activity of T and B lymphocytes, monocytes, natural killer cells, and immunoactive cytokines (interferon [IFN]-γ, interleukin [IL]-2, IL-6, and IL-12), providing a promising treatment for cancer patients^5^.

6-Sulfatoxymelatonin (aMT6s) is the primary urinary metabolite of melatonin. It is suggested that urinary aMT6s levels remain stable when sample processing is delayed for 24–48 hours^11^, and that urinary aMT6s in the morning is not influenced by sleeping pattern or by the storage time of urine, so it is selected to be a biomarker of plasma melatonin concentrations at the collection time^12^. As melatonin is mainly secreted at night, and its peak concentration occurs in the early morning, the detection of the first morning urinary aMT6s 12 hours overnight to assess the peak melatonin production is reliable^13, 14^. However, according to the secretion pattern of melatonin, levels of aMT6s collected at random time might not be useful as surrogates of nocturnal melatonin secretion. Although melatonin amplitudes show great variability, its range within the same person remains relatively stable, justifying singular melatonin measurements^13^. For our analysis, studies measuring the concentration of aMT6s in the first morning urine and 12-hour overnight samples were included.

Recent studies^15–22^ concerning the relationship of urinary aMT6s and BC provided mixed results. Travis *et al*.^13^ conducted the first prospective study implying no association between aMT6s and BC incidence. While Shernhammer *et al*.^16–18^ demonstrated a significantly inverse association between aMT6s level and BC risk, the remaining papers^20–22^ supported no evidence of such a negative association. To the best of our knowledge, up till now there is only 1 meta-analysis^23^ concerning this issue, which is however considered biased due to methodological dissonances and incomplete literature retrieval. Basler *et al*.^23^ found a weak but statistically significant inverse association between the urinary aMT6s level and BC risk (RR = 0.82, 95% CI, 0.68–0.99, *P* = 0.04) based on only 5 studies^13, 16–19^. While several factors (*e.g*., menopausal status and lag time) may influence the final conclusion potentially causing bias, with 5 studies Basler *et al*.^23^ could not do any subgroup analysis to provide the complete information. In addition, Basler *et al*.^23^ only searched one electronic database, suggesting an incomprehensive retrieval. Thus, their conclusions that melatonin affects BC incidence in women should be interpreted with caution and needs to be tested with the 3 emerging studies^20–22^. Herein an up-dated meta-analysis and systematic review was conducted to further assess the possible relationship between melatonin and the risk of BC based on studies investigating the urinary aMT6s concentrations.

## Results

### Study Selection

**Figure 1:**
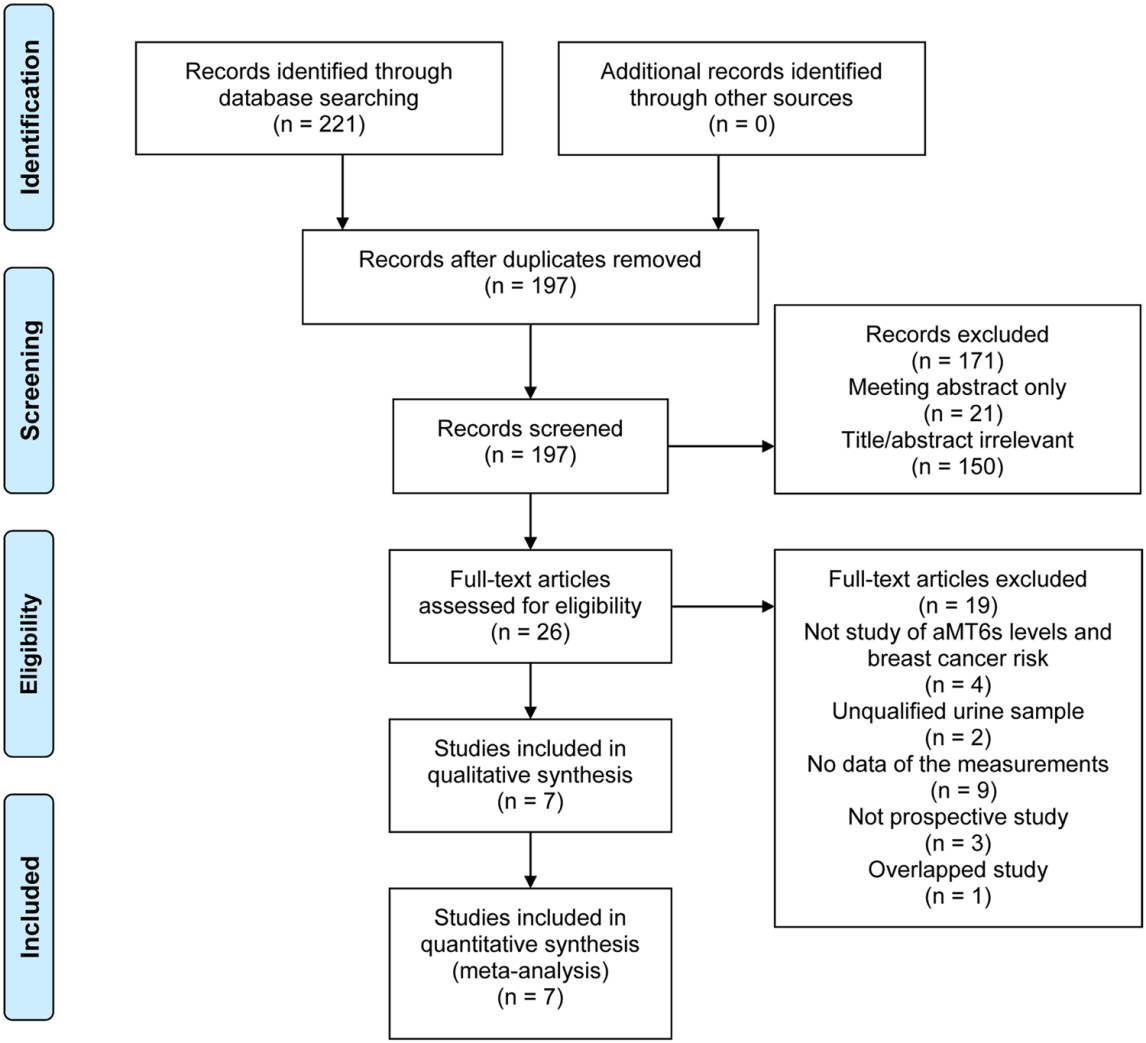
Literature retrieval flow diagram. aMT6s, 6-sulfatoxymelatonin.

A total of 26 studies were retrieved from the electronic databases PubMed, Cochrane Library, EMBASE, and Web of Science for full review during primary search according to the inclusion criteria, and 7 prospective case-control publications^16–22^ including 6 distinct studies^17–22^ were finally selected (Fig. 1). Wu *et al*.^24^ carried out a prospective trial on Chinese women in 2013, but the urine samples for measuring aMT6s were randomly collected not following a pre-specified time schedule (*e.g*., first morning and 12-hour overnight). As melatonin is mostly secreted during nighttime, the level of aMT6s in spot urine void could not reflect the real melatonin secretion. So this study was excluded. Studies carried out by Travis *et al*.^13^ and Wang *et al*.^21^ were both based on the Guernsey III Study with the same enrollment time. In Travis *et al*.’s study^13^, the end follow-up date was October 31^st^ 2001, while Wang *et al*.’s observation^21^ lasted until October 31^st^ 2009. Due to the overlap of the same group of participants, we selected Wang *et al*.’s study^21^ only for our meta-analyses. Likewise, Shernhammer *et al*.’s^16^ and Brown *et al*.’s studies^22^ included participants both from the NHSII cohort, and they followed them up to May 31^st^ 2001 and June 1^st^ 2007, respectively. To avoid selecting the same participants twice, Brown *et al*.’s study^22^ with a larger number of participants was included for the overall analysis. But for subgroup analyses, data from the Schernhammer *et al*.’s study^16^ were included if they were useful and were not overlapped. Furthermore, although Schernhammer *et al*.^17^ and Schernhammer *et al*.^19^ reported results from the same study cohort (the ORDET cohort), they investigated participants with different menopausal statuses. Therefore there was no dispute when selecting both studies^17, 19^. The characteristics of each study were listed in Table 1.

**Table 1:** Characteristics of the included studies in this meta-analysis.

Authors	Year	Data	Urine sample	Menopausal status	Country, cohort, and enrollment period
Schernhammer *et al*.	2005	Quartiles	1st morning urine	Pre- and post-menopausal	USA, NHSII cohort (1989), 1996–1999
Schernhammer *et al*.	2008	Quartiles	12 h overnight urine	Post-menopausal	Italy, ORDET cohort (post-menopausal), 1987–1992
Schernhammer *et al*.	2009	Quartiles	1st morning urine	Post-menopausal	USA, NHS cohort (1976), 2000–2002
Schernhammer *et al*.	2010	Quartiles	12 h overnight urine	Pre-menopausal	Italy, ORDET cohort, 1987–1992
Sturgeon *et al*.	2014	Quartiles	1st morning urine	Post-menopausal	USA, WHI cohort, 1993–1998
Wang *et al*.	2014	Tertiles	1st morning urine	Pre- and post-menopausal	Island of Guernsey, Guernsey III Study, 1977–1985
Brown *et al*.	2015	Quartiles	1st morning urine	Pre- and post-menopausal	USA, NHSII cohort (1989), 1996–1999

NHSII, Nurses’ Health Study II; NHS, Nurses’ Health Study; WHI, Women’s Health Initiative Observational; ORDET, Hormones and Diet in the Etiology of Breast Cancer Risk.

### Overall Analysis

**Figure 2:**
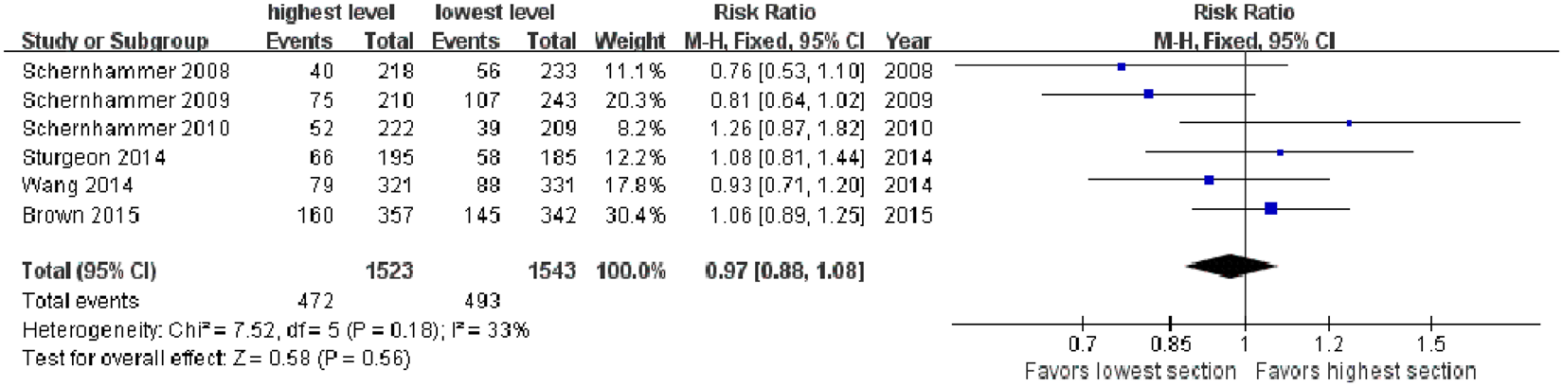
Forest plot for the association between urinary 6-sulfatoxymelatonin levels and breast cancer risk. Case subjects are defined as women who developed breast cancer after their enrollment in the study cohort, and matched healthy control subjects are randomly chosen, alive, and free of cancer at the time of diagnosis of the index case subject. Events indicate cases in the highest proportion or in the lowest proportion. Urinary 6-sulfatoxymelatonin levels are not significantly associated with overall breast cancer incidence.

The detailed highest and lowest levels in each study were defined in Table 2. Altogether, this meta-analysis included the overall data from 1824 women with incident BC and 3954 matched control participants in 6 studies^17–22^. The aMT6s levels in the BC and healthy control groups were listed in Table 3. When results from all the 6 distinct studies^17–22^ were pooled, the aggregate RR for BC was 0.97 (95% CI, 0.88–1.08; *Z* = 0.58; *P* = 0.56, Fig. 2), comparing women in the highest level of aMT6s concentration versus women in the lowest level, with no significant heterogeneity in estimates between the studies.

**Table 2:** The definition of highest and lowest levels in the included studies.

Study	Highest level (ng/mg creatinine)	Lowest level (ng/mg creatinine)
Schernhammer *et al*.^16^	≥28.8	<11.4
Schernhammer *et al*.^17^	≥16.5	<6.5
Schernhammer *et al*.^18^	≥34.3	<10.2
Schernhammer *et al*.^19^	≥20.6	<10.1
Sturgeon *et al*.^20^	≥22.2	<6.7
Wang *et al*.^21^ Overall	>20.4	<10.8
Wang *et al*.^21^ Pre-menopausal	>21.7	<12.1
Wang *et al*.^21^ Post-menopausal	>17.0	<8.3
Brown *et al*.^22^	≥61.9	<26.6

**Table 3:** Urinary 6-sulfatoxymelatonin concentration.

Study	Menopausal status	Group	Urinary 6-sulfatoxymelatonin concentration (ng/mg creatinine)	n	95% confidence interval
Schernhammer *et al*.^16^	Pre and post	BC	10.8	147	NR
		HC	12.7	291	NR
Schernhammer *et al*.^17^	Post	BC	21.0 (1.10)	178	NR
		HC	23.5 (0.55)	710	NR
Schernhammer *et al*.^18^	Post	BC	24.5 (29)	357	NR
		HC	28.8 (39)	533	NR
Schernhammer *et al*.^19^	Pre	BC	29.3 (1.11)	180	NR
		HC	27.6 (0.57)	683	NR
Sturgeon *et al*.^20^	Post	BC	16.3 (11.9)	258	NR
		HC	16.1 (12.9)	515	NR
Wang *et al*.^21^	Pre and post	BC	12.9	251	11.6–14.2
		HC	13.1	727	12.4–13.9
	pre	BC	14.6	160	13.0–16.4
		HC	14.6	465	13.6–15.6
	post	BC	10.6	62	8.5–13.3
		HC	10.7	179	9.4–12.2
Brown *et al*.^22^	Pre and post	BC	48.9 (31.8)	600	NR
		HC	47.9 (29.6)	786	NR

BC, breast cancer; HC, healthy control; NR, not reported.

### Subgroup Analyses

Subgroup analyses were conducted for pre- and post-menopausal patients, for invasive and *in situ* BCs, for lag time shorter and longer than 4 years, for estrogen receptor-positive (ER^+^) and ER^−^ BCs, and for studies using different urine samples (Table 4).

**Table 4:** Case number in the highest and lowest levels in the breast cancer and healthy control groups.

Category and study	Breast cancer			Healthy control		
	Highest	lowest	n	highest	lowest	n
Invasive breast cancer						
Schernhammer *et al*.^17^	37	55	171	173	171	683
Schernhammer *et al*.^18^	59	82	278	103	110	414
Brown *et al*.^22^	110	103	422	129	138	551
*In situ* breast cancer						
Schernhammer *et al*.^17^	3	1	7	5	6	27
Schernhammer *et al*.^18^	16	25	79	32	26	119
Brown *et al*.^22^	45	36	159	60	48	193
Lag time ≤ 4 y						
Schernhammer *et al*.^16^	36	61	147	94	94	291
Schernhammer *et al*.^19^	24	15	76	102	117	422
Sturgeon *et al*.^20^	21	17	80	36	46	160
Lag time > 4 y						
Schernhammer *et al*.^19^	28	24	104	68	53	261
Sturgeon *et al*.^20^	37	38	159	80	74	317
1^st^ morning urine						
Schernhammer *et al*.^18^	75	107	357	135	136	533
Sturgeon *et al*.^20^	66	58	258	129	127	515
Wang *et al*.^21^	79	88	251	242	243	727
Brown *et al*.^22^	160	145	600	197	197	786
12-hour overnight urine						
Schernhammer *et al*.^17^	40	56	178	178	177	710
Schernhammer *et al*.^19^	52	39	180	170	170	683

### Menopausal status

**Figure 3:**
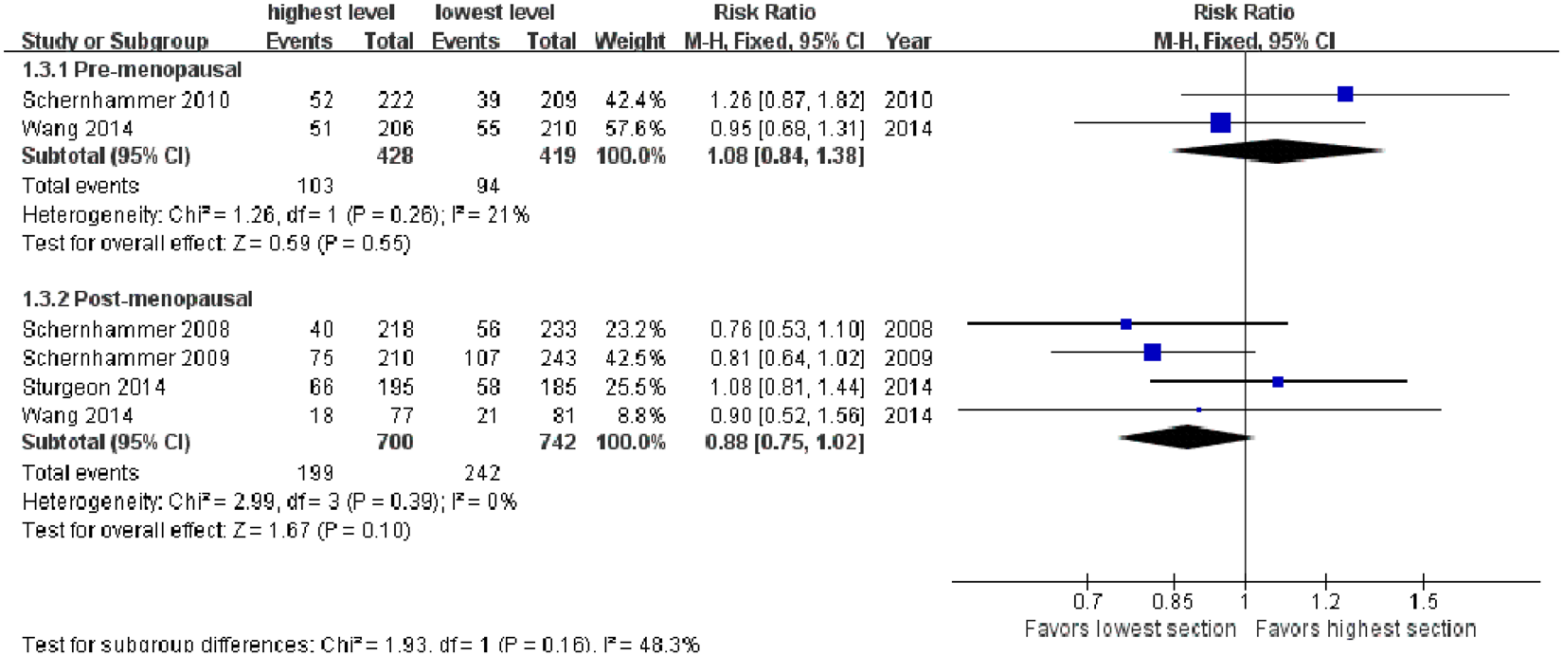
Forest plot for the association between urinary 6-sulfatoxymelatonin levels and breast cancer risk in pre- (upper sub-figure) and post-menopausal (lower sub-figure) women. 6-sulfatoxymelatonin levels are not significantly associated with breast cancer risk among premenopausal participants; while in postmenopausal women, there is an inverse association between 6-sulfatoxymelatonin level and tumor risk with a borderline significance.

aMT6s levels were not significantly associated with BC risk among premenopausal participants (highest level *vs*. lowest level, aggregate RR = 1.08, 95% CI, 0.84–1.38, *Z* = 0.59, *P* = 0.55), based on data from 2 studies^19, 21^. While in postmenopausal women, there was an inverse association between aMT6s level and BC risk with a borderline significance (RR = 0.88, 95% CI, 0.75–1.02, *Z* = 1.67, *P* = 0.10) based on data from 4 studies^17, 18, 20, 21^ (Fig. 3).

### BC pathological type

**Figure 4:**
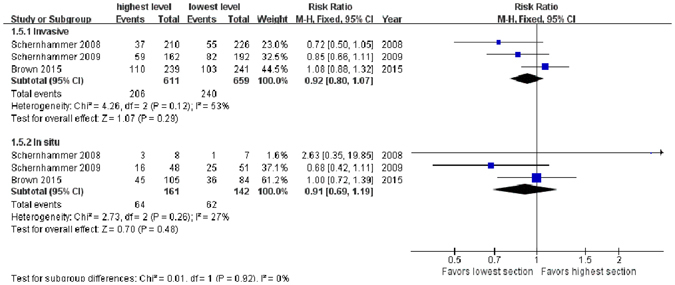
Forest plot for the association between urinary 6-sulfatoxymelatonin levels and risk of invasive (upper sub-figure) and *in situ* (lower sub-figure) breast tumors. There exists no significant association between 6-sulfatoxymelatonin level and risks of invasive or *in situ* breast cancer.

Three studies^17, 18, 22^ reported results for invasive and *in situ* BCs separately. The meta-analysis for aMT6s levels and invasive BC showed no association, and the RR for highest level *vs*. lowest level was 0.92 (95% CI, 0.80–1.07, *Z* = 1.07, *P* = 0.29), based on a fixed-effect model due to insignificant heterogeneity. The result for aMT6s and *in situ* BC was also insignificant, and the RR for highest level *vs*. lowest level was 0.91 (95% CI, 0.69–1.19, *Z* = 0.70, *P* = 0.48) (Fig. 4B).

### Lag time

**Figure 5:**
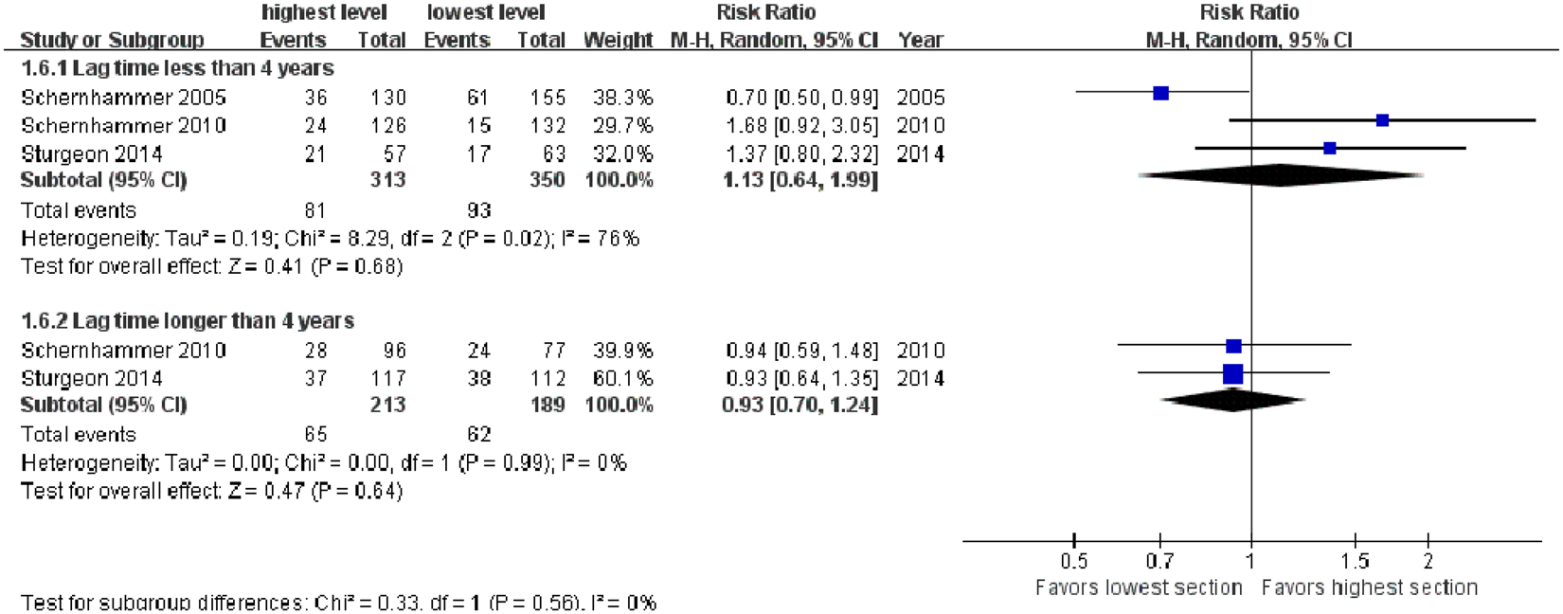
Forest plot for the association between urinary 6-sulfatoxymelatonin levels and breast cancer risk with lag time of less (upper sub-figure) and longer (lower sub-figure) than 4 years. For both kinds of lag time, no significant associations are detected.

As the lag time differed within the selected studies^15–22^, we pooled data to find out whether different lag time influenced the final result. For lag time of less than 4 years, aMT6s levels were not associated with BC risk (highest level *vs*. lowest level, RR = 1.13, 95% CI, 0.64–1.99, *Z* = 0.41, *P* = 0.68, 3 studies^16, 19, 21^), using a random-effects model due to significant heterogeneity (*χ* ^*2*^ = 8.29, *P* = 0.02, *I* ^*2*^ = 76%). A similar result was seen in the subgroup where lag time was longer than 4 years (highest level *vs*. lowest level, RR = 0.93, 95% CI: 0.70–1.24, *Z* = 0.47, *P* = 0.64, 2 studies^19, 20^) (Fig. 5).

### ER expression

**Figure 6:**
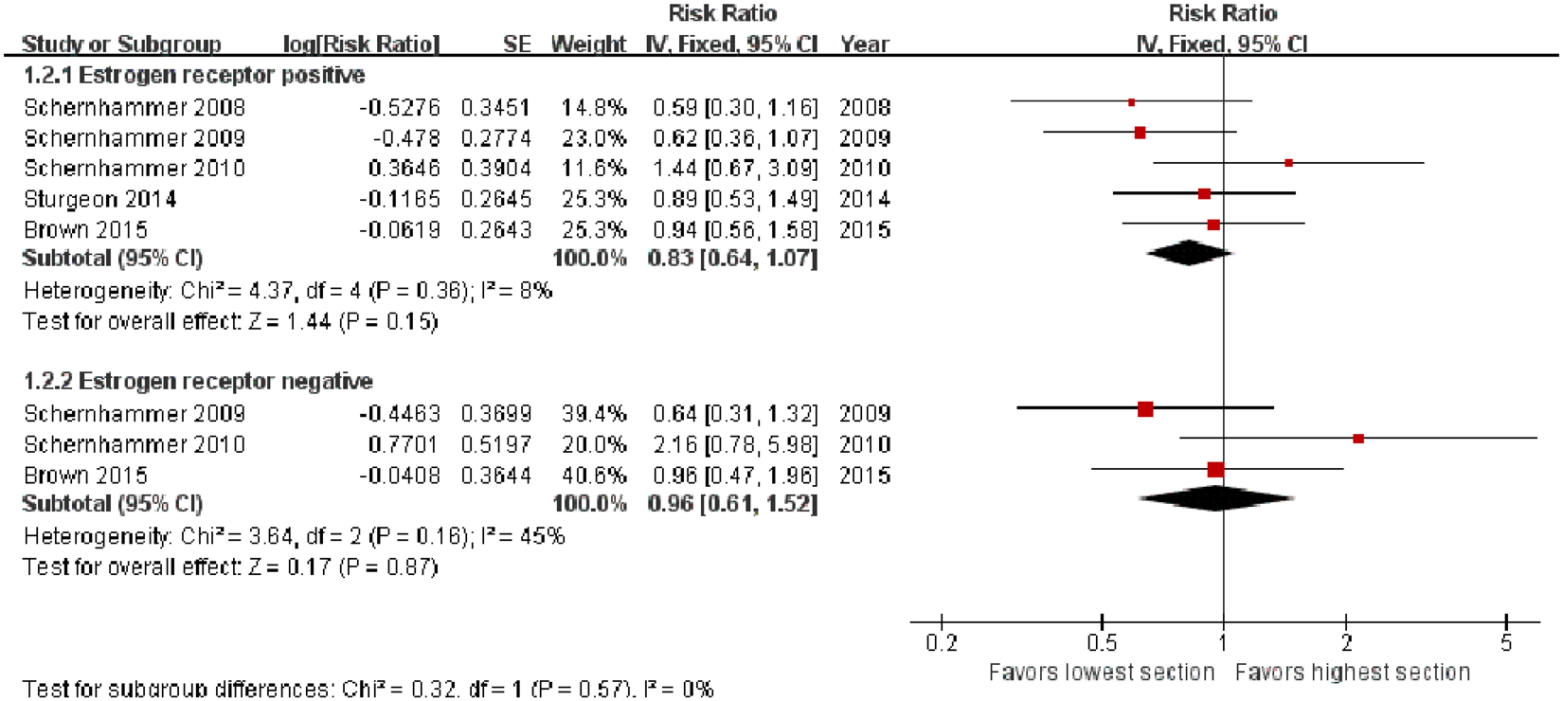
Forest plot for the association between urinary 6-sulfatoxymelatonin levels and estrogen receptor positive (upper sub-figure) and negative (lower sub-figure) breast cancer risk. Urinary 6-sulfatoxymelatonin is inversely associated with estrogen receptor positive breast cancer with a borderline significance, but not with estrogen receptor negative tumors.

When we restricted the analysis to women with ER^+^ BC, a negative association between aMT6s and BC risk with a borderline significance was shown (highest level *vs*. lowest level, RR = 0.83, 95% CI, 0.64–1.07, *Z* = 1.44, *P* = 0.15, 5 studies^17–20, 22^). However, the risk of ER^−^ BC was not significantly lower in participants with the highest level of aMT6s (highest level *vs*. lowest level, RR = 0.96, 95% CI: 0.61–1.52, *Z* = 0.17, *P* = 0.87, 3 studies^18, 19, 22^) (Fig. 6).

### Urinary sample

**Figure 7:**
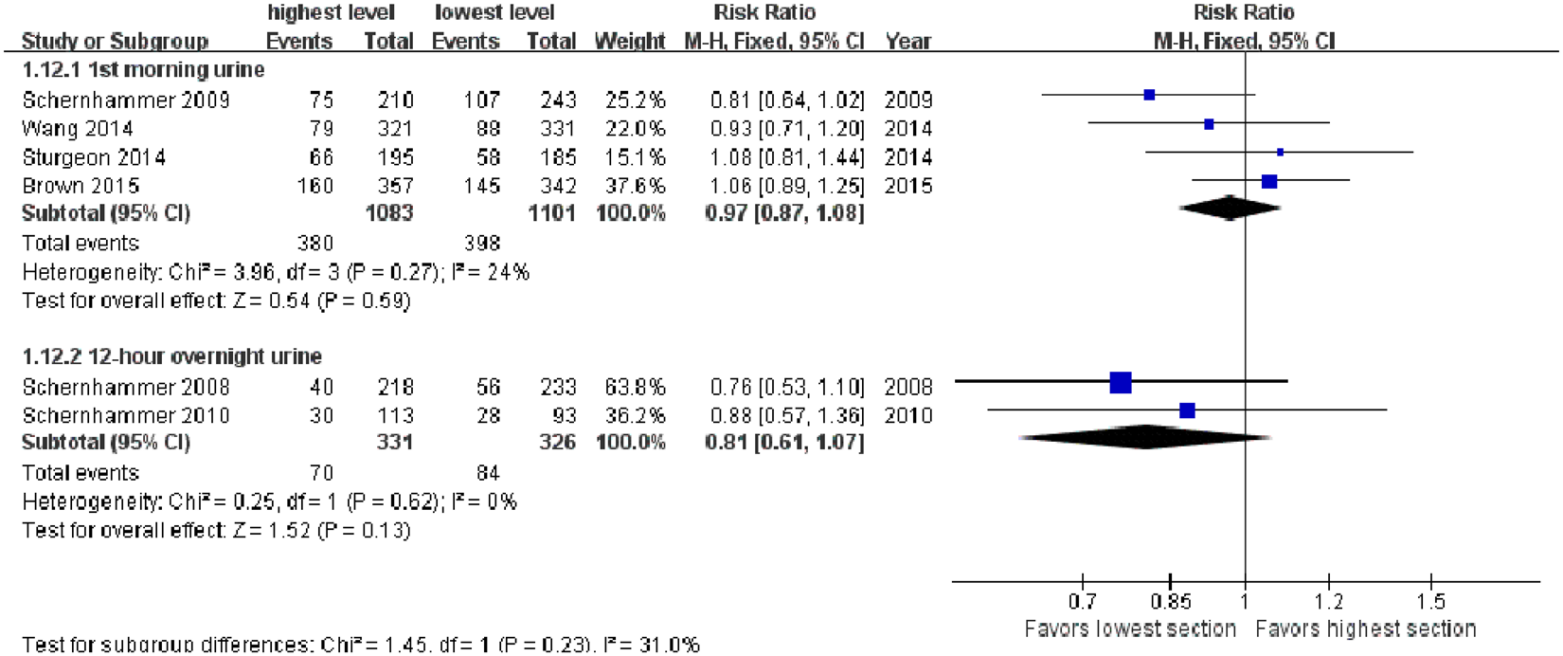
Forest plot for the association between urinary 6-sulfatoxymelatonin levels in first morning (upper sub-figure) and 12-hour overnight (lower sub-figure) urine samples and breast cancer risk.

The meta-analysis based on the 4 studies^18, 20–22^ using first morning urinary samples indicated no link between aMT6s and BC incidence (highest level *vs*. lowest level, RR = 0.97, 95% CI, 0.87–1.08, *Z* = 0.54, *P* = 0.59). On the contrary, for the 12-hour overnight urine samples, aMT6s was negatively associated with BC risk with a borderline significance (highest level *vs*. lowest level, RR = 0.81, 95% CI, 0.61–1.07, Z = 1.52, *P = *0.13, 2 studies^17, 19^) (Fig. 7).

### Bias Assessment

We performed funnel plot analysis and Egger’s test to evaluate the potential bias. Judging from the linear regression test of funnel plot asymmetry, it was suggested that the data were distributed evenly. The result from the Egger’s test showed that there was no indication of a bias for this meta-analysis (*P* = 0.412). Funnel plots further supported that there were not any significant biases. (Supplementary Figure 1)

## Discussion

A total of 7 publications^16–22^ including 6 distinct studies^17–22^ that met the inclusion criteria were selected, and the evidence extracted from them was summarized quantitatively. To our surprise, there was no evidence of statistical association between urinary aMT6s and BC risk, which was inconsistent with the previous reports^21, 23^. Clinical investigations suggested an inverse association between nocturnal plasma melatonin level and the incidence of BC^25–27^. The meta-analysis performed by Basler *et al*.^23^ demonstrated a significant inverse association between aMT6s level and BC risk. And Yang *et al*.^28^ implied that an increase of 15 ng/mg creatinine in aMT6s reduced BC risk (RR = 0.86, 95% CI, 0.78–0.95), with a significant linear dose-response trend. In this analysis, we revealed no significant link between aMT6s and BC risk (RR = 0.97, *P* = 0.56). One of the possible explanations might be that melatonin may co-function with many other factors during breast carcinogenesis. Artificial light at night may induce melatonin suppression and vitamin D insufficiency, leading to BC^29^. And shiftwork was suggested to have a significant association with BC risk^30^ with reduction of 25-hydroxyvitamin D concentration^31^ and vitamin D deficiency^32^. Since low melatonin and low 25OHD concentration are both the results of night shiftwork, it is suggested that melatonin is not the only significant risk reduction factor for BC. Further studies investigating the association between urinary aMT6s and BC incidence should take these factors into consideration.

Of all the studies included, although Schernhammer was the first author in 4 included articles^16–19^, their data sets are distinct from each other due to discrepant cohorts or characteristics of participants. Schernhammer *et al*.^17^ and Schernhammer *et al*.^19^ selected premenopausal and postmenopausal participants respectively from the ORDET cohort. While Schernhammer *et al*.’ study^16^ was based on the NHSII cohort, and Schernhammer *et al*.’ study^18^ originated from the NHS cohort. For the studies based on the same cohorts^15, 16, 21, 22^, overlaps of study recruitment period was observed. The studies^21, 22^ with the most participants and longest follow-up period were included. Besides, useful data from Schernhammer *et al*.^16^ were included in subset analyses. Therefore there is no dispute about initially selecting all these 7 studies.

In 2014, Basler *et al*.^23^ performed a meta-analysis and found a negative association between the urinary melatonin level and BC risk. The electronic database searched by Basler *et al*.^23^ was only PubMed from 1989 to 2013. As meta-analysis requires a comprehensive, objective, and reproducible search of a range of sources to identify as many relevant studies as possible, we enlarged the retrieval using PubMed, Cochrane Library, EMBASE, and Web of Science and identified additional studies^21^, suggesting an inadequate retrieval by Basler *et al*.^23^. Furthermore, as melatonin secretion is affected by various factors, such as menopausal status^33, 34^ and some preclinical diseases, their meta-analysis was not sufficient without taking these factors into consideration. After that, 3 novel studies^20–22^ with a larger sample size were published, and the results of these papers were inconsistent with Basler *et al*.’s findings^23^. Compared with the former meta-analysis^23^, our meta-analyses had several strengths. First, more studies with a larger number of participants were included. Markedly more subgroup analyses were carried out. Second, all the included studies had high qualities according to the good research design and matched control selection criteria^35^. Third, no limitation was set during literature search, potentially decreasing selection bias. In addition, the 3 newly enrolled studies^20–22^ had significantly enlarged sample size, and 2 of them^21, 22^ could cover the previous reports with improved quality.

It was unclear whether methodological dissonances (some of the studies^18, 20–22^ use first morning urine samples and others^17, 19^ 12-hour overnight samples) led to mixed results of all the included studies. So we carried out a subgroup analysis based upon diverse urine samples. Interestingly, pooled results from studies^18, 20–22^ using first morning urine samples showed no association between aMT6s and BC incidence, while an inverse association with a borderline significance was observed when we restricted samples to 12-hour overnight urine (RR = 0.81, 95% CI, 0.61–1.07). As many postmenopausal women may void during the night, so ‘first morning’ urine may not reflect a true overnight first void^20^. On the contrary, 12-hour overnight urine samples might well mirror the melatonin secretion. However this finding needs to be treated with caution as only 2 studies^17, 19^ were included.

Upon different cut-points of these studies, aMT6s concentrations were classified into quartiles or tertiles. The division of aMT6s was based on the distribution in the controls. In our study, the relative BC risks of the highest and lowest quartile (tertile respectively) were determined, compared, and used to calculate the corresponding odds ratios. All of the 6 studies^17–22^ used creatinine-adjust aMT6s to reflect the concentration of urinary aMT6s. As urinary creatinine concentration is influenced by many factors, including sex, ethnicity, and age, the mean aMT6s concentration varied.

Some of the identified studies^17, 19, 20, 22^ highlighted the lag time between urine collection and BC diagnosis, because of the possibility of preclinical tumors affecting urinary aMT6s levels. Schernhammer *et al*.^17, 19^ tested the trend of the association between aMT6s and BC occurrence with increasing lag time. They excluded cases that were diagnosed shortly after urine collection, using a stepwise approach. The association between urinary aMT6s level and breast cancer risk became increasingly inverse after excluding case patients who were diagnosed with invasive breast cancer within 2 years (OR for highest versus lowest quartile 0.68), 4 years (OR = 0.61), or 8 years after urine collection (OR = 0.17). However, the lag time analyses conducted by Sturgeon *et al*.^20^ suggested no significantly decreased risks of BC with higher urinary levels of melatonin when restricting analyses to those with lagged exposure by 4 or more years after urinary collection. Similar conclusion was also drawn by Brown *et al*.^22^. To address this question, we performed analysis based on 3 studies^14, 17, 19^ which provided data of lag intervals of less than 4 years, and the RR for BC risk on highest level *vs*. lowest level was insignificant. When we restricted the lag time to longer than 4 years^17, 18^, the RR was also insignificant. Therefore no trend of stronger association between aMT6s level and BC was observed with a longer lag interval. However, as a limited number of studies was included in this subgroup analysis, further studies with larger participants and various lag time are need for more in-depth investigation.

Although no overall link between aMT6s and BC incidence was elucidated, there existed an inverse association between aMT6s level and ER^+^ BC incidence with a borderline significance (RR = 0.83, 95% CI, 0.64–1.07). The mechanisms underlying melatonin’s protection against ER^+^ BC are becoming clearer^36^. Melatonin works through receptors and distinct second messenger pathways^33, 37^ to reduce cellular proliferation and to induce cellular differentiation. A physiological peak nighttime serum value of melatonin could delay and slow tumor progression via interfering with the malignant cell cycle, suppressing the proliferation of ER^+^ human BC cell lines significantly and directly^38^. Melatonin interferes with the estrogen-signaling pathways^39^, by suppressing the ERα mRNA expression and the estrogen-induced transcriptional activity. Besides, melatonin impacts the expression of growth inhibitory and apoptotic pathway modulators including TGF-α, Bax, and CaM^39^. Schernhammer *et al*.^15–17^ found that the significantly inverse association between aMT6s levels and tumor incidence remained when only ER^+^ BC was considered, which was moderately in accordance with the previous findings^8, 29, 30^. Likewise, Chottanapund *et al*.^8^ demonstrated melatonin as an aromatase inhibitor in the co-culture system. In 2001, Hansen *et al*.^40^ found that night work and melatonin were more strongly related to invasive than *in situ* BC risk. Similar results were presented by Thompson and Li^41^ investigating melatonin and invasive BC. The association was limited to postmenopausal women. In a study exploring levels of melatonin and sex hormones^11^, although melatonin appeared to be not directly associated with recent night work and estrogen levels, long-term night work did seem to increase the estrogen levels among postmenopausal women. These were in half agreement with our findings that there presented an inverse association between aMT6s levels and postmenopausal BC incidence, although with borderline significance (RR = 0.88, *P* = 0.10). However, we observed no association of aMT6s and invasive BC incidence. These call for further investigations due to the relatively limited number of studies in these subgroups.

This study is limited by the diverse classifications of the aMT6s levels, the different definition of lag time, the various urine collection ways, and the discrepant primary menopause statuses. And due to the limited number of included studies and some heterogeneities, our analyses should also be interpreted with caution. Besides, as many other potential confounding factors have been suggested to be associated with BC incidence, such as vitamin D, 25OHD, artificial light at night and shiftwork^29, 30, 32^, further studies need to take all these factors into consideration. Particularly, among the included studies, there is only one by Schernhammer *et al*.^16^ reporting the influence of night shiftwork. Although the corresponding subgroup analysis might not be possible in this case, the study found that results remained similar after excluding participants just having the night shift. Moreover, our analyses were majorly based on specific classifications of the aMT6s concentration only available in included studies, future investigations quantitatively defining BC-associated aMT6s concentrations might be warranted.

In conclusion, our meta-analysis showed that there was no significant association between the levels of urinary aMT6s and the risk of BC, while an inverse association with a borderline significance was observed in postmenopausal women and ER^+^ BC patients. The role of aMT6s in predicting BC risk might require further investigations. As the public interest has increasingly focused on the potential morbid risk of the light-at-night work schedules and the circadian disruption with emphasis on melatonin^42^, additional studies with further explorations are needed to validate the association between aMT6s/melatonin and BC risk.

## Materials and Methods

### Publication Search

This meta-analysis was guided by the Preferred Reported Items for Systematic Reviews and Meta-Analysis (PRISMA) statement issued in 2009^43^. The electronic databases PubMed, Cochrane Library, EMBASE, and Web of Science were searched for relevant published studies up to November 6^th^ 2016, using the following keywords: “melatonin/6-sulfatoxymelatonin/6-sulphatoxymelatonin” and “breast/mammary cancer/carcinoma”. The American Society of Clinical Oncology annual meeting abstracts have also been retrieved.

### Inclusion Criteria

To be considered eligible for our meta-analysis, the relevant studies were carefully selected based on the following criteria: (1) available baseline status of enrolled women; (2) prospective studies; (3) BC incidence in relation to highest levels and lowest levels of first morning and 12-hour overnight urinary aMT6s; and (4) risk ratio (RR)/odds risk (OR) reported with a 95% confidence interval (95% CI).

Xu J and Huang L implemented the literature search, and identified eligible papers according to the inclusion criteria. In case of discrepancy, consensus was reached through discussion with Sun GP’s participation. Multiple articles covering the same research were identified. And for those overlapping publications, only the most recent publication or the one with the largest number of participants, most abundant information, and longest follow-up period was included. Data from the overlapping studies, if useful for subgroup analyses, were included as well where appropriate.

### Data Extraction and Definition

Data extraction and quality assessment were conducted by Xu J and Huang L. The data extracted from each eligible study included authors’ names, years, study design, baseline characteristics, concentration of aMT6s (quartiles or tertiles), and urine samples. Case subjects were defined as women who developed BC after their enrollment in the study cohort, and matched healthy control subjects were randomly chosen, alive, and free of cancer at the time of diagnosis of the index case subject. Levels of urinary aMT6s remain stable when processing is delayed for 24–48 hours, so aMT6s resolution after sample collection is not a matter of concern^13^. Besides, the levels of aMT6s in first-spot morning and 12-hour overnight urine samples are both moderately associated with melatonin secretion^12, 14, 44^, suggesting them as reliable study samples. Lag time was defined as the interval from a participant’s enrollment to the diagnosis of BC. Concerning the urinary aMT6s levels, the first quantile corresponded to the lowest level, and the highest quantile to the highest level, as specified by each study.

### Statistical Analysis

RRs were used to estimate the association between urinary aMT6s concentration and BC incidence. The 95% CIs were further calculated. The Mantel-Haenszel’s method was applied for meta-analyzing dichotomous results, and the Inverse Variance strategy was used for pooling RRs in the overall analysis as well as in the subgroup analyses based on ER status. The *χ* ^*2*^-based *Q*-test (*P* < 0.05 was considered significant) was applied to calculate the heterogeneity or the *I* ^*2*^ statistic was used to examine the extent of cross-study heterogeneity. Data were analyzed using the fixed-effect or the random-effects model based on data heterogeneity^45^. For analyses using the random-effects model, the Tau2 test^46^ was also performed to indicate heterogeneity. Funnel plot was drawn and Egger’s test^47^ was carried out to investigate potential publication bias. Sensitivity analyses were applied to estimate the influence of individual study on the overall effect. All statistical analyses were performed using RevMan 5.3 and Stata 11. All *P* values were two-sided.

## Electronic supplementary material

Supplementary Information
