# Supplementary material for: Urinary 6-sulfatoxymelatonin level and breast cancer risk: systematic review and meta-analysis
Source: Sci Rep. 2017 Jul 13;7:5353. doi: 10.1038/s41598-017-05752-9 (PMC5509698; doi:10.1038/s41598-017-05752-9)
Supplement: Supplementary file 1 — Supplementary Information [file 41598_2017_5752_MOESM1_ESM.pdf]

Title: Urinary 6-sulfatoxymelatonin level and breast cancer risk: systematic review and meta-analysis

Authors: Jing Xu*, Lei Huang*, and Guo-Ping Sun

* Jing Xu and Lei Huang contributed equally to this work.

Supplementary Figures

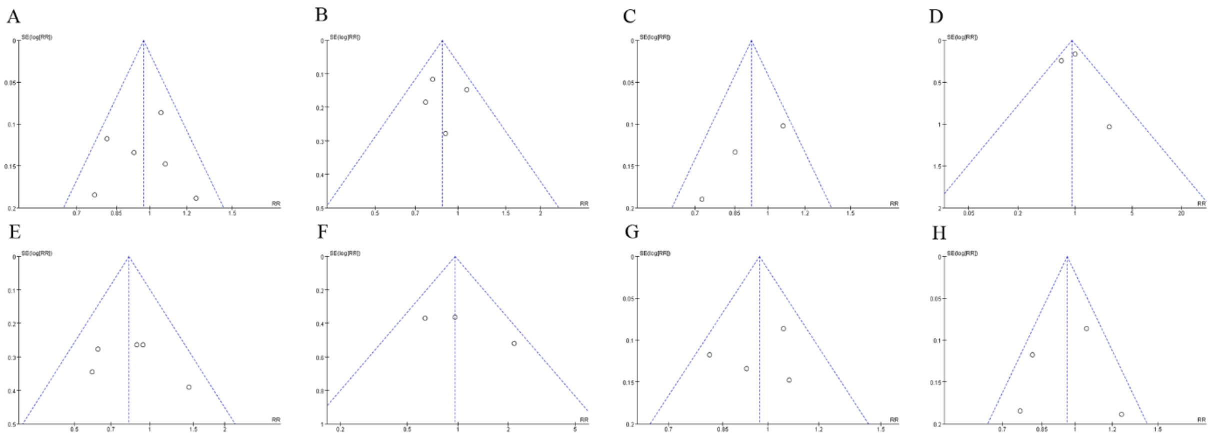

Supplementary Figure 1. Funnel plots for the overall correlation (A), correlations between urinary 6-sulfatoxymelatonin and postmenopausal (B), invasive (C), *in situ* (D), estrogen receptor positive (E), and estrogen receptor negative (F) breast cancers, and correlation using the first morning urine (G) and from studies by the Schernhammer group (H). All indicate insignificant biases.
